# Design and experimental validation of a metamaterial-based sensor for microwave imaging in breast, lung, and brain cancer detection

**DOI:** 10.1038/s41598-024-67103-9

**Published:** 2024-07-13

**Authors:** Musa N. Hamza, Slawomir Koziel, Anna Pietrenko-Dabrowska

**Affiliations:** 1 Department of Physics, College of Science, University of Raparin, Sulaymaniyah, 46012 Iraq; 2 Engineering Optimization & Modeling Center, Reykjavik University, 102 Reykjavik, Iceland; 3 Faculty of Electronics, Telecommunications and Informatics, Gdansk University of Technology, 80-233 Gdansk, Poland

**Keywords:** Microwave imaging (MWI), High-gain antenna, Metamaterials (MTMs), Artificial magnetic conductor (AMC), Cancer diagnosis, Electrical and electronic engineering, Applied physics, Biological physics, Imaging techniques, Design, synthesis and processing, Breast cancer, Cancer screening, Lung cancer, Sensors and biosensors

## Abstract

This study proposes an innovative geometry of a microstrip sensor for high-resolution microwave imaging (MWI). The main intended application of the sensor is early detection of breast, lung, and brain cancer. The proposed design consists of a microstrip patch antenna fed by a coplanar waveguide with a metamaterial (MTM) layer-based lens implemented on the back side, and an artificial magnetic conductor (AMC) realized on as a separate layer. The analysis of the AMC’s permeability and permittivity demonstrate that the structure exhibits negative epsilon (ENG) qualities near the antenna resonance point. In addition, reflectivity, transmittance, and absorption are also studied. The sensor prototype has been manufactures using the FR4 laminate. Excellent electrical and field characteristics of the structure are confirmed through experimental validation. At the resonance frequency of 4.56 GHz, the realized gain reaches 8.5 dBi, with 3.8 dBi gain enhancement contributed by the AMC. The suitability of the presented sensor for detecting brain tumors, lung cancer, and breast cancer has been corroborated through extensive simulation-based experiments performed using the MWI system model, which employs four copies of the proposed sensor, as well as the breast, lung, and brain phantoms. As demonstrated, the directional radiation pattern and enhanced gain of the sensor enable precise tumor size discrimination. The proposed sensor offers competitive performance in comparison the state-of-the-art sensors described in the recent literature, especially with respect to as gain, pattern directivity, and impedance matching, all being critical for MWI.

## Introduction

Cancer belongs to the most dangerous diseases affecting humanity. Estimations indicate that by 2030 there would be 17 million cancer-related deaths and 26 million new cancer diagnoses yearly^1^. Gene alterations that promote abnormal development and tumor formation that can spread to other parts of the body are the cause of breast cancer^2,3^. The most frequent cancer in women^2,3^, the number of cases is expected to rise from 14 to 22 million over the course of the next 20 years^4,5^. Breast cancer may be successfully treated most of the time following an early detection (99% survival rate)^6,7^. On the other hand, the most frequent kind of cancer in the world in terms of new occurrences is lung cancer. The main risk factor for lung cancer, which causes 80% to 90% of cases, is tobacco smoking. 2.1 million new cases of cancer were recorded in 2018, making about 12% of all cases globally. Males accounted for the majority of diagnoses in 2018, with over 1.37 million cases. The regions with the greatest occurrence rates were Eastern Asia, Polynesia, Central and Eastern Europe, and Micronesia. The incidence rate is lower in women; in 2018, about 725,000 new cases were diagnosed. In 2020, lung cancer accounted for 1.8 million new fatalities, or 18% of all deaths linked to cancer. The 5-year survival rate for lung cancer is lower than that of other severe types of cancer^8^. In the United States, lung cancer is the second most common cancer among both men and women, after the breast cancer. It is most commonly diagnosed in men after the prostate cancer^9^. Medical classification of the sickness include many phases based on the size of the tumor and the location of aberrant cell proliferation throughout the human body. The early stages of lung cancer are characterized by a small tumor that has not yet spread to any lymph nodes. Identification of lung cancer in its early stages enhances treatment results and prevents the malignant tissues from spreading^10,11^. Brain cancer is another extremely severe kind of the disease. One clear definition of brain tumors, which are thought to be among the most complex types of tumors, is the growth of tissue with abnormal cell development^12^. Unchecked brain tumor growth can result in brain cancer, which is currently the ninth leading cause of death worldwide and is predicted to climb in the future^13^. The two main categories of brain tumors are benign and malignant^14^. The National Brain Tumor Society (NBTS) projects that by 2022, over 90,000 Americans will have received a primary brain tumor diagnosis. Brain tumors are on the rise worldwide. More over 25,000 of the tumors are malignant, while over 600,000 are benign^15^. Malignant tumors have an irregular shape and a variable structure, whereas benign tumors are consistently formed. Malignant tumors develop uncontrolled and cause greater rates of death than benign tumors, which grow slowly. Prompt diagnosis, vigilant monitoring, and appropriate investigation can reduce mortality and improve the survival rates^16,17^.

To detect breast, lung, or brain cancer, modern healthcare facilities use a variety of imaging techniques, including mammography, X-ray screening, computed tomography (CT) scanning, positron emission tomography (PET), biopsies, ultrasound screening, and magnetic resonance imaging (MRI)^18–22^. The imaging modalities that radiologists and doctors today have access to enable early diagnosis of diseases including breast cancer, lung cancer, and brain tumors. The main drawbacks of these techniques include high radiation doses that increase the risk of cancer, the risks to foetuses and elderly patients, the ionization of brain cells, high cost, the risks to pacemaker and implanted cardioverter patients, the length of the diagnostic process, and the decreased willingness of otherwise healthy people to undergo screening due to the possible risks involved with the procedure^23–26^. MWI has recently drawn a lot of attention in the context of medical applications due to its potentially remarkable qualities, which include non-ionizing radioactivity, penetration capability at low power, non-invasiveness, no risk of ionization for the human body, cost-effectiveness, as well as low profile (hardware-wise)^27^. The inside architecture of the human body may be seen with microwave imaging, which makes use of electromagnetic fields operating at microwave frequencies between 300 MHz and 30 GHz. Microwave imaging may be realized using three methods: hybrid, active, and passive^28^. Active MWI creates microwave pictures by first delivering microwave impulses into the tissues via sensors, then accumulating the signal reflections. Active microwave imaging techniques, such as tomography and radar imaging, are employed for a range of purposes, including non-invasive breast cancer detection. Ultra-wideband (UWB) radar imaging is required for the latter^29,30^. Similar techniques can be used to identify lung cancer and brain cancer^31^. The most often used are simple ones, such as radar-based, where signal reflections captured at different locations may be used to identify the strong scattering region^32^. The main components of a MWI system are an image processing unit, mechanical parts, and an antenna array. The antenna is an important piece of hardware, and the picture it produces depends greatly on its properties. The microwave signals are transmitted to the region of interest by a single antenna, and the backscattered signals are detected by one or more receiving antennas. When an antenna is utilized in MWI systems, its gain, radiation pattern, and impedance bandwidth are its three most crucial performance metrics. These factors have an impact on MWI's sensitivity and accuracy in detecting cancerous cells. The physical size and form of the antenna are also important to minimize interference and increase the signal-to-noise ratio for accurate readings.

The following are some of the antenna solutions that have been proposed for the detection of breast cancer: CPW-fed monopole antenna^33^, side-slotted Vivaldi antenna^34^, CPW-fed EBG-based antenna^35^, rectangular slotted patch antenna^36^, semi-circle shaped planar antenna^37^, antipodal Vivaldi antenna^38^, antipodal Vivaldi antenna^39^, side slotted Vivaldi antenna^40^, and slotted antipodal Vivaldi antenna^41^. A number of antennas, elliptic patch UWB antenna^42^, including slot-rotated antenna^43^, coaxial antenna^44^, and two horn-type and Vivaldi-type antennas used as the transmitter and receiver, respectively^45^, have been proposed for the development of a microwave lung imaging (MLI) system for the detection of lung tumors. Furthermore, a variety of antennas have been proposed for use in designing an MBI system that is intended to detect brain tumors. These include, among others, a 3D stacked folded antenna^46^, a wideband monopole antenna^47^, a conformal wideband antenna^48^, a metamaterial-loaded stacked antenna array^49^, an antipodal Vivaldi antenna^43^, an EBG-based microstrip patch^45^, a 3D-slot-loaded folded dipole^42^, a 3D stacked wideband antenna^44^, a bowtie antenna^50^, a cross-fed 3D slot-loaded antenna^51^, and a GCPW-based slotted inverted delta shaped patch^52^. A careful review of the literature reveals that the main objective behind most contemporary designs, including the antenna configurations discussed above, was to maximize the impedance bandwidth. Other aspects, such as radiation quality and gain, have not been given priority in terms of their improvement. However, as previously mentioned, both the electrical (impedance matching) and field performance (gain, radiation patterns) are essential for the MWI systems to provide high-quality diagnostics. Moreover, the design strategies and particular antenna configurations that have been documented in the literature were developed with the aim of identifying a specific kind of malignancy, and demonstrated accordingly. However, the development of reliable, multifunctional, high-resolution screening instruments that medical professionals can rely on requires the application of significantly enhanced sensors.

This article presents a novel microstrip sensor designed for high-resolution microwave imaging (MWI) for early detection of breast, lung, and brain cancer. The sensor consists of a microstrip patch antenna fed by a coplanar waveguide with a metamaterial-layer-based lens, and an artificial magnetic conductor (AMC) involving a perfect reflector. The AMC exhibits negative epsilon qualities near the antenna resonance point, and its electrical and field characteristics are confirmed through experimental validation. The sensor's suitability for detecting brain tumors, lung cancer, and breast cancer was demonstrated through extensive simulation testing with the MWI system. The directional radiation pattern and enhanced gain enable precise tumor size discrimination. The proposed sensor offers competitive performance over the state-of-the-art devices reported in the literature, especially in terms of gain, pattern directivity, and impedance matching, all of which are critical for high-quality MWI.

## Proposed sensor design: antenna and AMC

**Figure 1:**
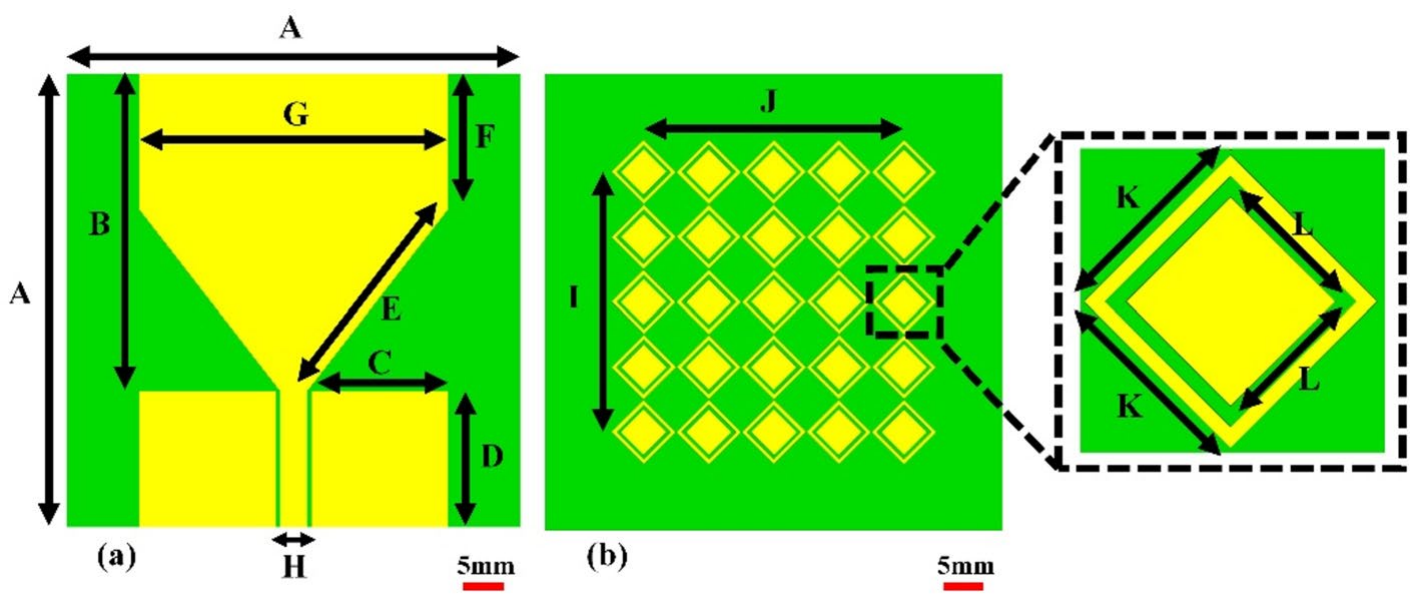
Proposed antenna: (**a**) top layer: microstrip patch, (**b**) bottom layer: MTMs layer's geometry, which includes an inset displaying the MTM unit cell.

**Figure 2:**
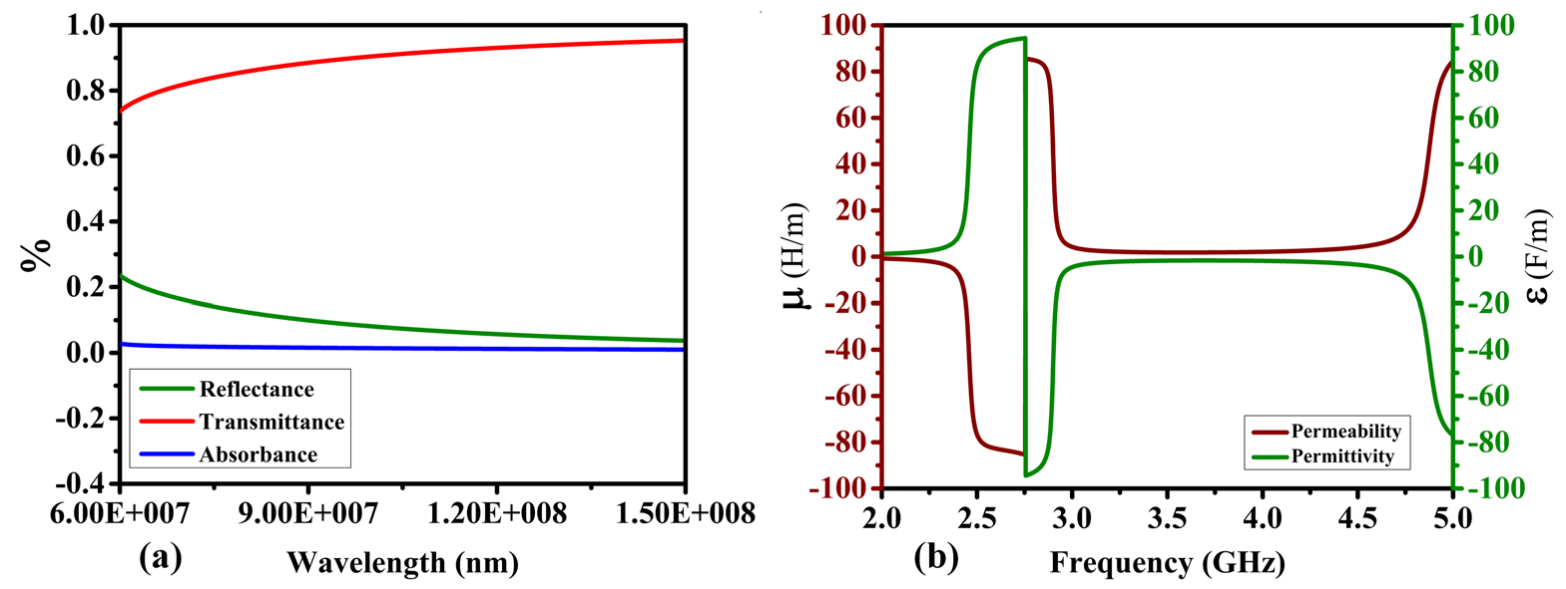
MTM characteristics: (**a**) transmittance, absorbance, and reflectance, (**b**) permeability and permittivity.

**Figure 3:**
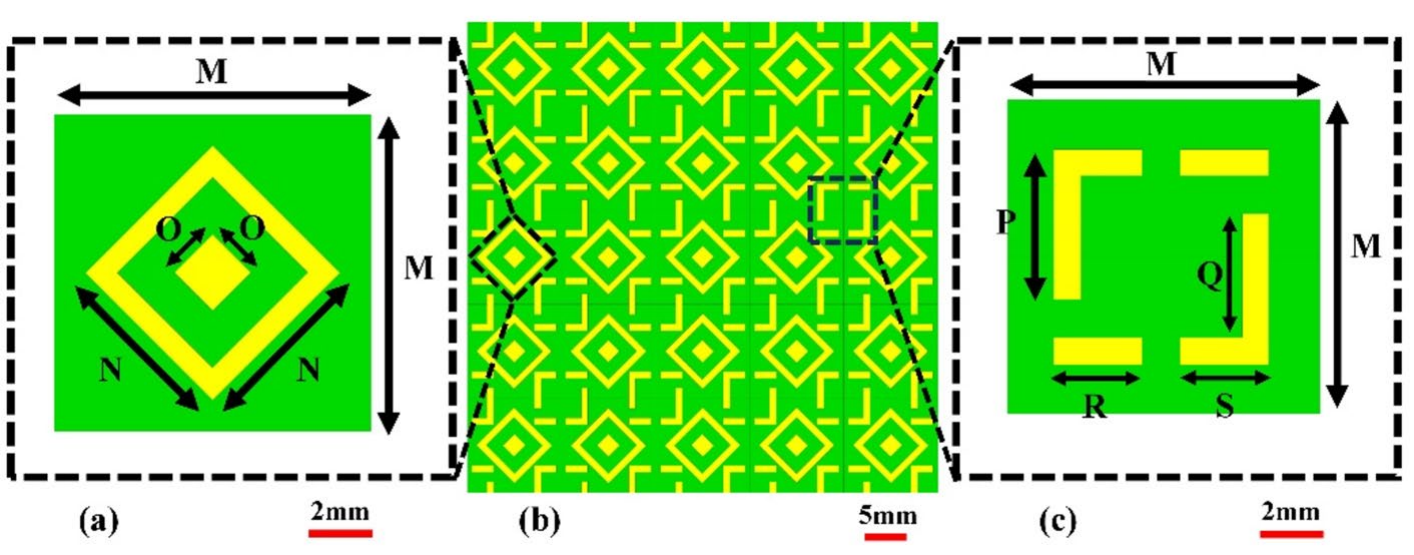
AMC geometry: (**a**) first MTM unit cell geometry; (**b**) front view geometry of the AMC structure; and (**c**) second MTM unit cell geometry.

**Figure 4:**
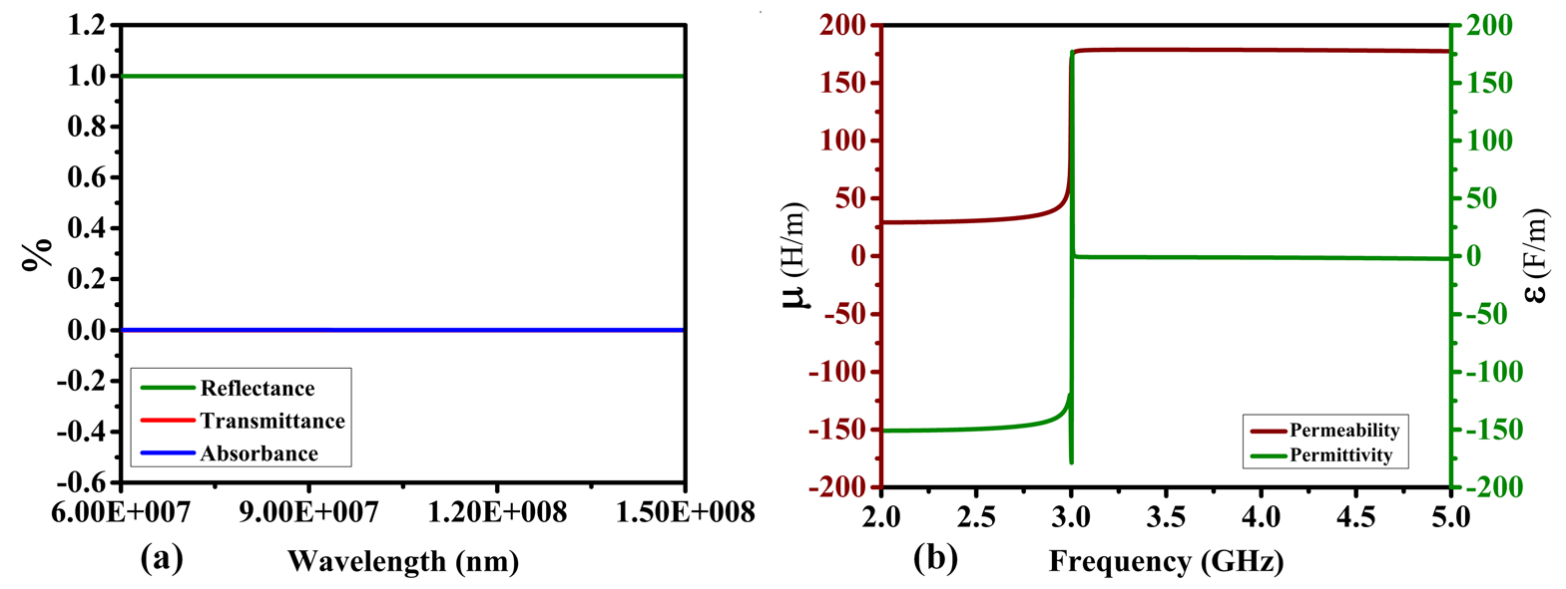
AMC characteristics: (**a**) transmittance**,** absorbance, and reflectance, (**b**) permeability and permittivity.

**Figure 5:**
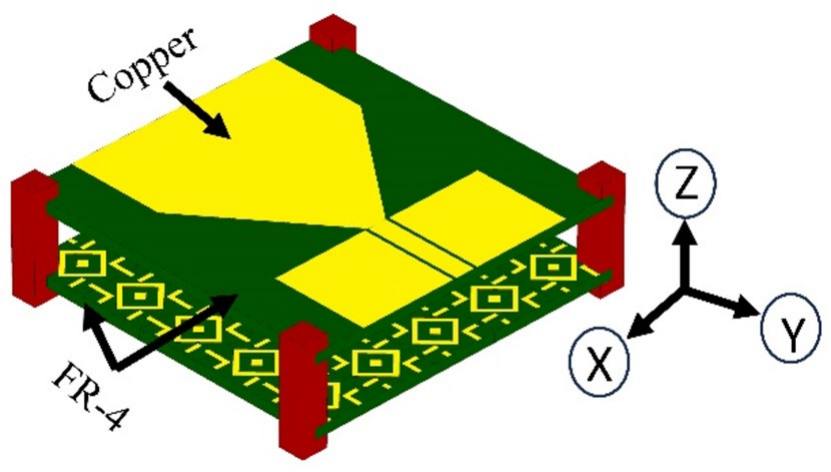
Complete geometry of the proposed sensor.

This section discusses the design process of the proposed sensor, including the microstrip patch antenna and the artificial magnetic conductor (AMC) structure. The antenna is implemented on a 1.52-mm-thick FR4 substrate with a relative permittivity of 4.3 and dimensions of 50 mm × 50 mm, respectively. Figure 1a shows the geometry of a basic patch antenna. The antenna is designed to operate within the 2 GHz to 5 GHz band, balancing penetration depth and resolution to facilitate cancerous cell detection. The experimental validation indicates that the effective operational bandwidth of the proposed antenna with AMC is approximately from 2.5 to 4.75 GHz, as confirmed by the return loss (∣*S*11∣) and gain measurements. This range is critical for achieving the desired gain and impedance matching, which are essential for high-quality MWI. Other important properties of the sensor from the point of view of microwave imaging are radiation patterns (which should be directive) as well as high gain. Here, to achieve required field properties, an artificial magnetic conductor (AMC) is proposed, implemented on an extra layer to be discussed in the next paragraph. At this point, it should be mentioned that while AMC improves the antenna performance from the point of view of features critical for MWI, it can also be a source of noise in the image. High power levels can lead to non-linear behavior, such as harmonic products and intermodulation. When building an AMC-coupled microwave imaging system, these considerations must be carefully taken into account. When various signals are present, the AMC can combine them, creating new frequencies by adding and subtracting the original frequencies and their harmonics. These nonlinear effects can have a severe influence on microwave imaging systems that use AMCs because the noise created by harmonics and intermodulation products can obscure the target signal, making it harder to distinguish and potentially leading to erroneous picture reconstruction. This issue can be mitigated by implementing a metamaterial array on the backside of the antenna, as shown in Fig. 1b. The proposed metamaterial acts as a perfect lens between the proposed antenna and the AMC, within the wavelength range from the 7⋅10^7^ nm to 1.5⋅10^8^ nm, showing a throughput of more than 80%, as explained in Fig. 2a. Additionally, the metamaterial is designed so that its permeability is positive and the permittivity is negative in the 2 GHz to 2.75 GHz band. However, in the band from 2.75 to 5 GHz, the permeability is negative and the permittivity is positive, as explained in Fig. 2b. One of the effects of the metamaterial is lowering the power level of the input signal, which is the most effective technique to reduce nonlinear effects. Also, customizing the power level based on the minimal power necessary for the desired imaging performance helps balance noise and signal strength. Furthermore, the metamaterial layer allows for concentrating the incoming waves and directing them towards the AMC's front surface. Following the orderly return of the waves to the antenna, the metamaterial sends the majority of the incoming waves to the antenna while allowing for minimum internal overlap between the antenna and the AMC. This minimizes the amount of noise created, resulting in higher-resolution photos. After examining the properties of the metamaterial, the AMC design, consisting of two different metamaterials, was proposed, as explained in Fig. 3b. The first type of unit cell consists of two concentric rhombuses arranged as a 5 × 5 array on a 50 × 50 mm^2^ FR4 substrate, as depicted in Fig. 3a. The second type of unit cell consists of an intermittent square also arranged as a 5 × 5 array, as shown in Fig. 3c. Using two different metamaterials provides more flexibility in terms of achieving the specific attributes and properties. Our sensor's metamaterial layer is intricately composed of artificially structured unit cells, combining dielectric and metallic elements in a carefully engineered arrangement. These unit cells are designed to create resonance effects, particularly at the microstrip patch antenna's resonance frequency. Through this design, the metamaterial layer exhibits negative permittivity and permeability, resulting in negative epsilon (ENG) characteristics crucial for microwave imaging applications. This configuration enhances the sensor's performance by enabling precise focusing, increased directivity, and heightened sensitivity to electromagnetic variations, essential for accurate and early detection of breast, lung, and brain abnormalities in medical diagnostics. The metamaterial's tailored electromagnetic interactions play a pivotal role in optimizing the sensor's capabilities, contributing significantly to its effectiveness in high-resolution microwave imaging for medical purposes. The back of the AMC is covered with copper material to eliminate transmitted waves, which significantly reduces noise in the microwave imaging process. The proposed AMC is designed to act as a perfect reflector, so the efficiency of the proposed AMC for incoming electromagnetic waves in the 2GHz to 5GHz band is approximately 99.9%**.** Also, the absorption and transmission capacity of the proposed AMC account for approximately 0.1%, as demonstrated in Fig. 4a. The proposed AMC has negative permittivity and positive permeability, as shown in Fig. 4b. This property is known as epsilon negative (ENG) in the classification of metamaterials. When AMC is designed as a perfect reflector and placed behind the antenna, it improves the antenna's performance by increasing radiation efficiency, decreasing backward radiation, and increasing the front-to-back ratio. By directing more electromagnetic energy in the appropriate direction, it not only refines the antenna's focus but also reduces interference. The following sections fully demonstrate the effects of AMC on antenna performance. The AMC's negative epsilon properties also contribute to this improvement. The negative epsilon AMC, in conjunction with a perfect magnetic conductor, offers unmatched manipulation of electromagnetic waves, providing enhanced impedance matching and superior antenna performance within the specified frequency bands. Figure 5 shows a perspective view of the complete sensor architecture. Table 1 provides information about the optimized system dimensions. The dimensions of our innovative microstrip sensor were meticulously determined through a systematic design process aimed at achieving optimal performance in microwave imaging (MWI) applications. While the final antenna size may appear larger than traditional starting estimates, this deliberate sizing was essential to incorporate advanced features such as the metamaterial layer-based lens and the artificial magnetic conductor (AMC) structure, both crucial for enhancing the sensor's imaging capabilities. The decision to exceed the typical half-wavelength size was driven by the need to achieve specific performance objectives, including high realized gain, directional radiation pattern, and wide impedance bandwidth, as outlined in the objectives of our study. This intentional sizing approach allowed us to realize the desired ENG characteristics near the antenna resonance point, contributing significantly to improved sensitivity and quality of medical tests using MWI. Furthermore, the enhanced gain and directional radiation pattern facilitated precise tumor size discrimination, a critical aspect in early cancer detection and treatment planning. While compactness is an important consideration in antenna design, especially for certain applications, our focus in this study was primarily on optimizing performance parameters essential for MWI-enabled cancer detection. The detailed analysis and validation of our sensor's electrical and field characteristics, including extensive simulation-based experiments and experimental validations, support the effectiveness and suitability of our design for real-world imaging applications. Optimizing our sensor design by exploiting dielectric property variations in cancerous tissues holds promise for enhancing detection accuracy. Detailed analysis allows us to tune the sensor to resonate at frequencies maximizing sensitivity and contrast for each cancer type, improving diagnostic accuracy. Integrating cancer biomarkers into the metamaterial unit cell design further enhances detection sensitivity and specificity. Fine-tuning the sensor's parameters, including antenna geometry, metamaterial composition, and AMC structure, aligns its electromagnetic characteristics with various cancer types. Validating these optimizations through simulations and experiments with tissue-mimicking phantoms is crucial. Collaboration with medical and oncology experts refines our sensor, tailoring it for specific diagnostic needs, ultimately advancing early cancer detection and patient outcomes.

**Table 1:** Optimized geometry parameters of the proposed sensor.

Parameter	Value (mm)	Parameter	Value (mm)	Parameter	Value (mm)
A	50	H	3	O	1.69
B	35	I	28.5	P	3.95
C	15	J	28.5	Q	3.24
D	15	K	5	R	2.33
E	25.3	L	3.54	S	2.33
F	15	M	10	Space	9
G	34	N	5.65		

## Simulation results

**Figure 6:**
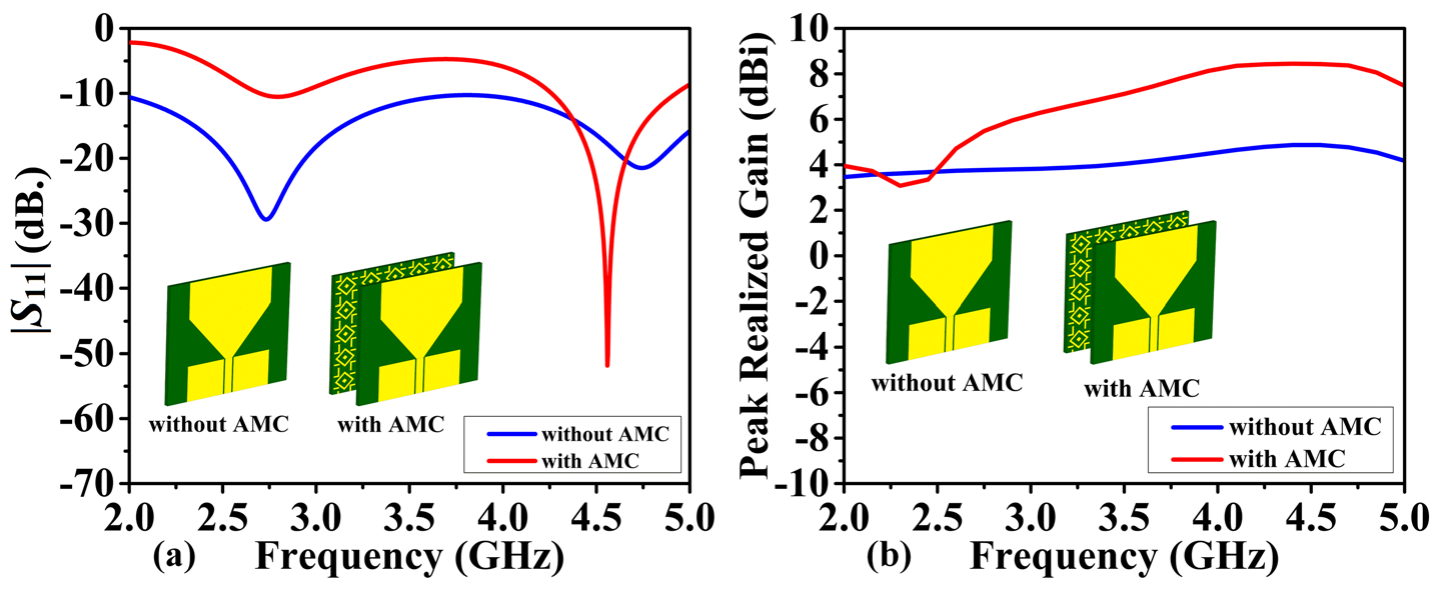
Proposed sensor, without and with AMC: (**a**) simulated reflection coefficient |*S*_11_|, (**b**) simulated realized gain.

Figure 6 displays the simulated reflection and realized gain responses of the proposed sensor with and without an AMC. As it can be observed, antenna resonances occur at 2.75 GHz and 4.75 GHz in the absence of the AMC, but at 4.562 GHz when the AMC is present, as shown in Fig. 6a. After integrating the AMC, the first resonance point (2.75 GHz) vanished, and the second resonance has been shifted from 4.75 to 4.562 GHz. These effects may be explained by the AMC's perfect reflection and negative permittivity working in synergy. At the rear of the antenna, the perfect reflection creates a new electrical boundary condition that modifies the current distribution and suppresses particular resonant modes, causing the initial resonance to vanish. Furthermore, negative permittivity changes the phase velocity of electromagnetic waves within the AMC structure, influencing the total effective length of the antenna and, as a result, altering the second resonance point. However, this shift is accompanied by an increased resonance depth as a result of the improved energy confinement and lower total losses contributed to by the AMC's perfect reflection. As a result, the observed variations in resonance behavior are the result of a complex interaction between the inherent features of the antenna and the electromagnetic environment provided by the AMC's specific functions.

**Figure 7:**
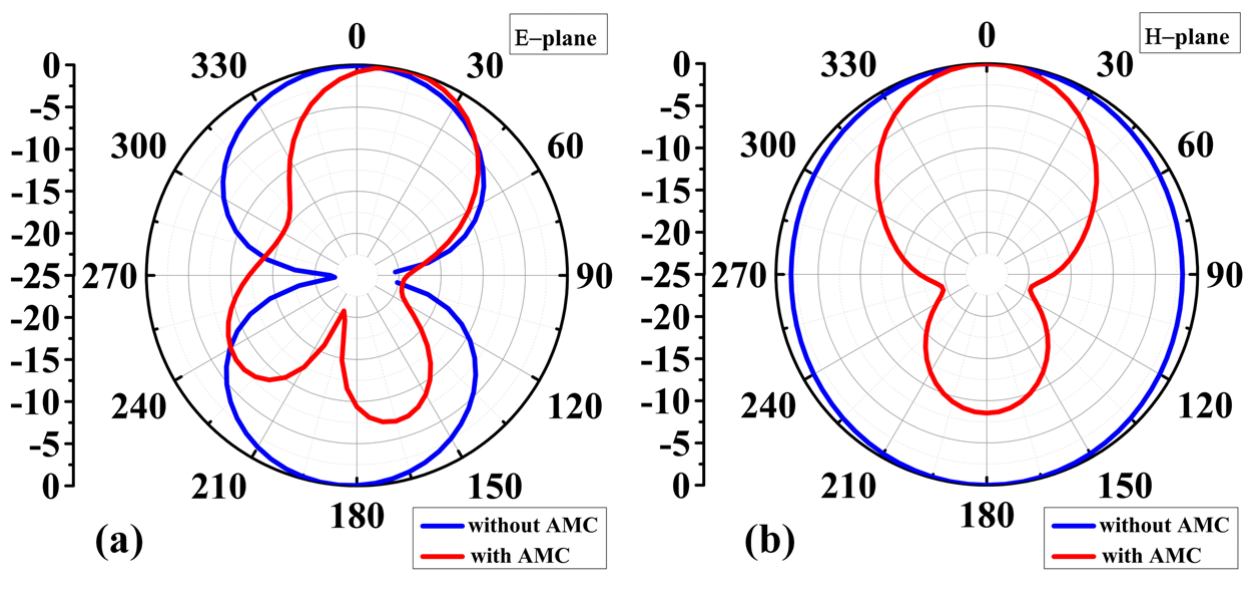
Sensor's normalized radiation pattern was simulated at resonance frequency, both with and without AMC: (**a**) E-plane, and (**b**) H-plane.

Figure 6 shows the peak realized gain of the proposed sensor. At the corresponding resonant frequencies, the realized gain with and without the AMC is 8.4 dBi and 4.8 dBi, respectively. Thus, the presence of AMC increases the gain by 3.6 dBi. Figure 7 shows the simulated 2D radiation patterns in the *xz*-(φ = 0) and *yz*-(φ = 90) planes, respectively. AMC reduces back radiation and focuses it on the broad side. At the resonant frequency, the almost perfect reflection of the incident electromagnetic wave by the AMC causes a significant change in the antenna radiation pattern. The antenna radiates in both directions (forward and backward). However, this behavior changes significantly upon attaching the AMC. The positive permeability and negative permeability of the AMC create a reflected surface with equal but opposite impedance to the impedance of the open space. This effectively reflects any waves that reach the antenna. Through constructive interference, these reflected waves merge with the forward propagating waves, greatly increasing their strength and creating a single dominant frontal lobe. At the same time, destructive interference between the reflected and incident waves reduces their amplitude, thereby reducing back radiation. This selective filtering improves gain and directivity, resulting in more focused and efficient transmission of electromagnetic waves. The data presented shows that the AMC layer improves all important antenna characteristics, including radiation pattern, gain, and impedance matching. Specifically, the AMC antenna has a unidirectional transmission pattern that improves signal quality, reduces interference and increases signal reception, contributing to more accurate and reliable images. Additionally, the use of high-gain antennas improves the clarity and resolution of images obtained by MWI, allowing for more precise diagnosis and treatment planning.

## Experimental validation

This section elaborates on experimental validation of the antenna, the AMC, and the complete sensor introduced in "Proposed sensor design: antenna and AMC" section. We start by discussing the fabrication methodology and experimental setup, followed by the presentation of the antenna and sensor characteristics (reflection response, gain, and radiation patterns). The section is concluded with the analysis of the obtained results.

### Antenna prototype

**Figure 8:**
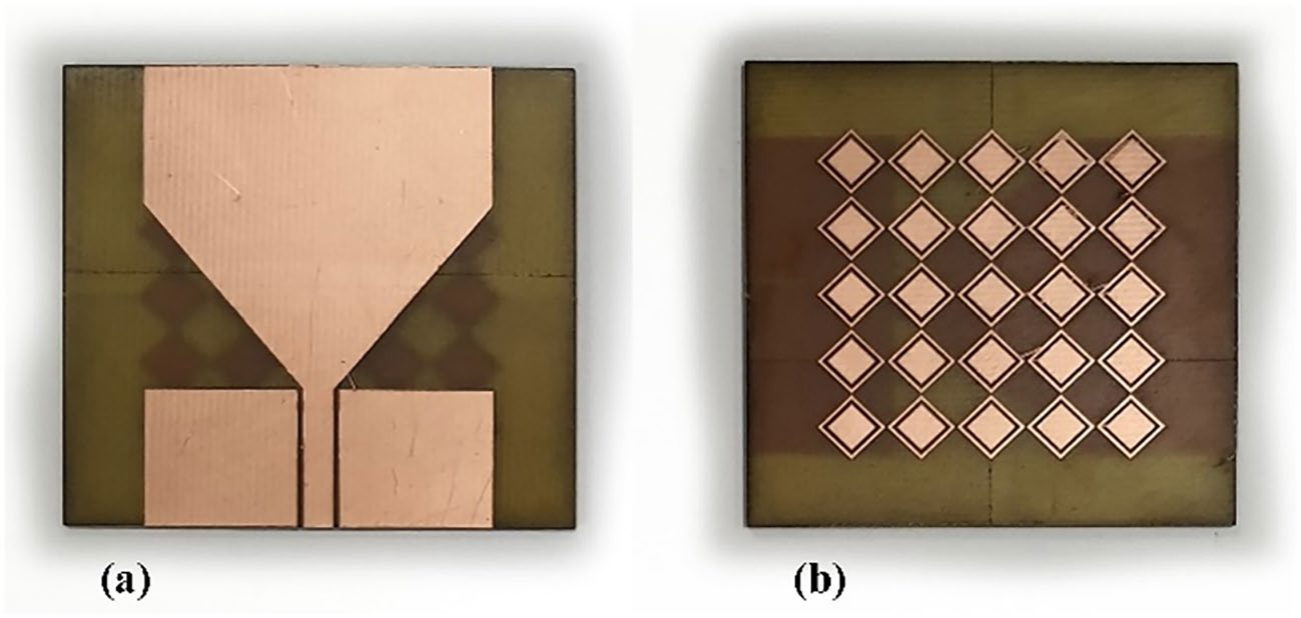
Antenna prototype photographs: (**a**) front view, (**b**) back view with MTMs design.

**Figure 9:**
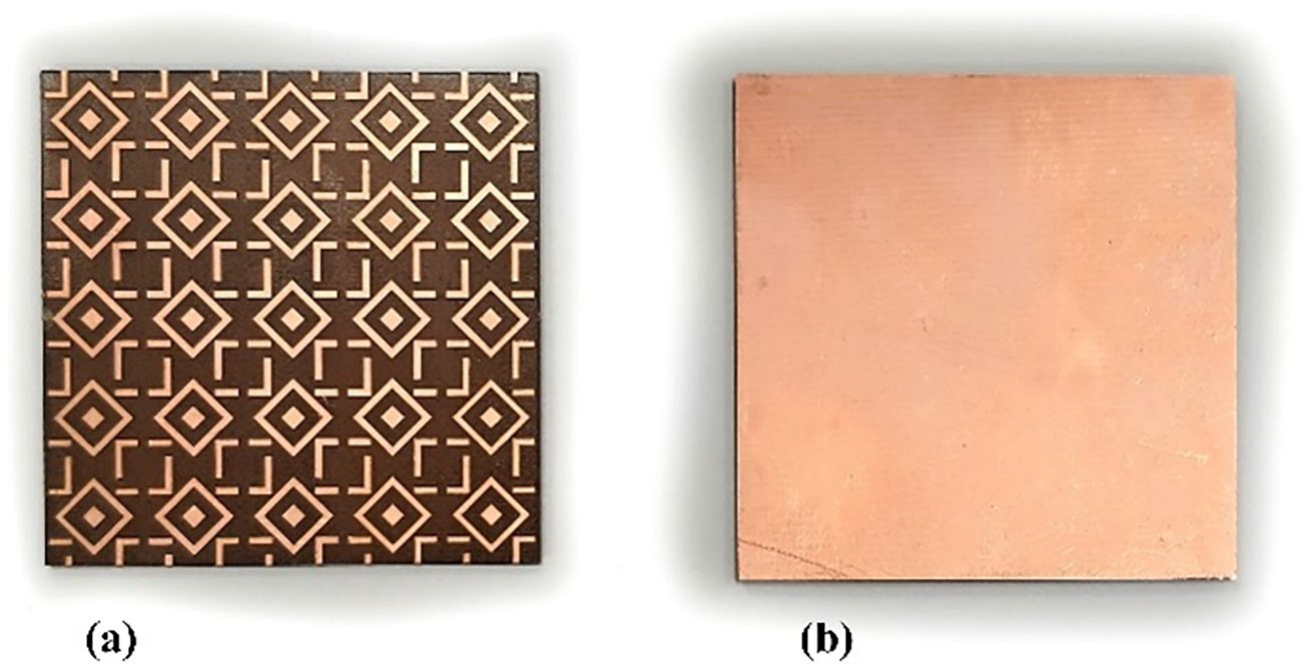
AMC prototype photographs: (**a**) front view, (**b**) rear view (full metal surface).

**Figure 10:**
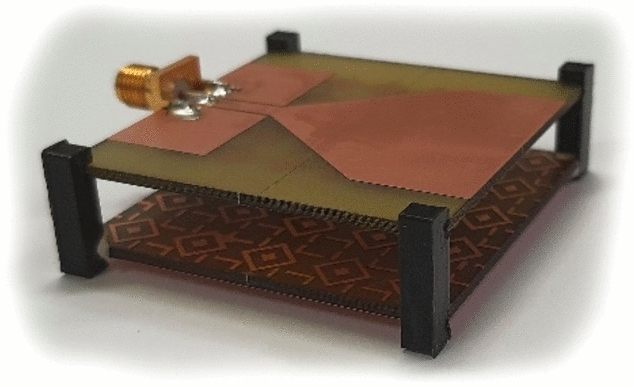
Perspective view of the entire sensor prototype.

The proposed antenna has been fabricated on a 1.52-mm-thick FR4 substrate using an LPKF protolaser machine. The antenna/AMC dimensions have been provided in Table 1. Figures 8 and 9 show photographs of the antenna and AMC prototypes, respectively. Figure 10 depicts a perspective view of the complete sensor.

### Experimental setup

**Figure 11:**
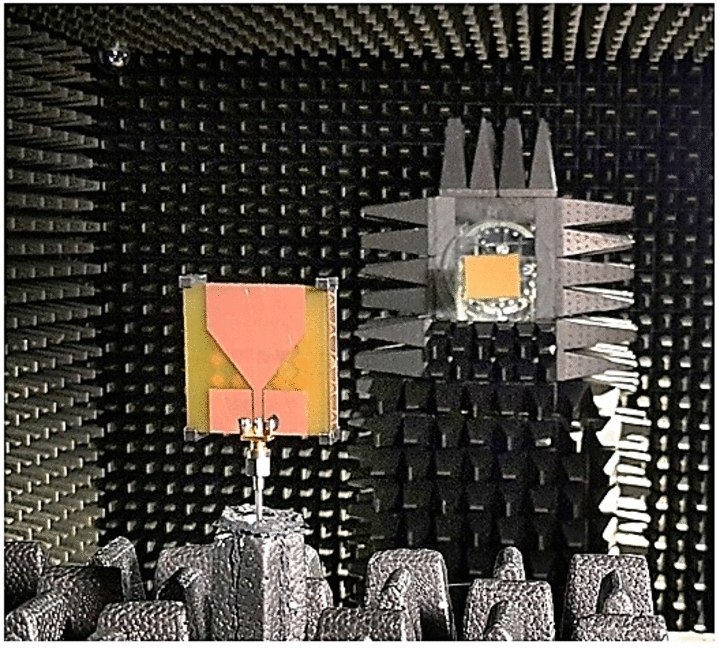
Experimental setup in the anechoic chamber.

Figure 11 shows the experimental setup. The measurements have been carried out using the anechoic chamber at Reykjavik University, Iceland, the 0–40 GHz Anritsu MS4644B vector network analyzer (VNA) and a Geozondas GZ0226DRH 2–26 GHz horn antenna.

### Experimental results

**Figure 12:**
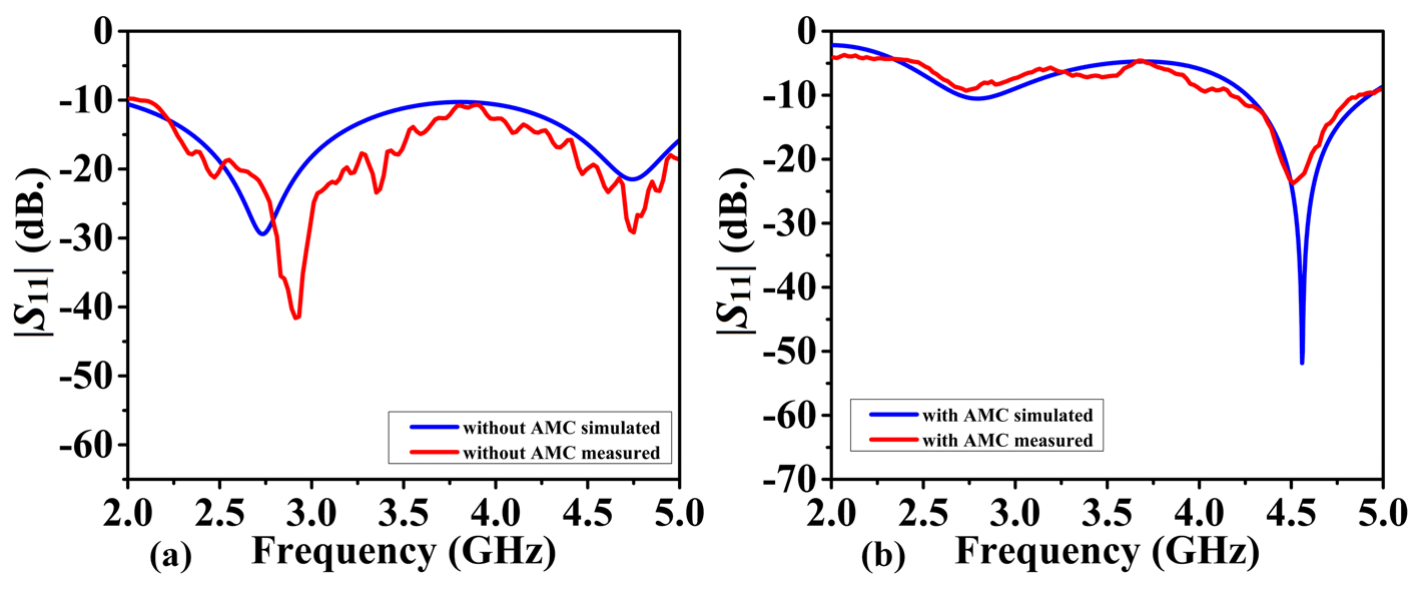
Reflection coefficient |*S*_11_| measured and simulated: (**a**) without AMC, and (**b**) with AMC.

**Figure 13:**
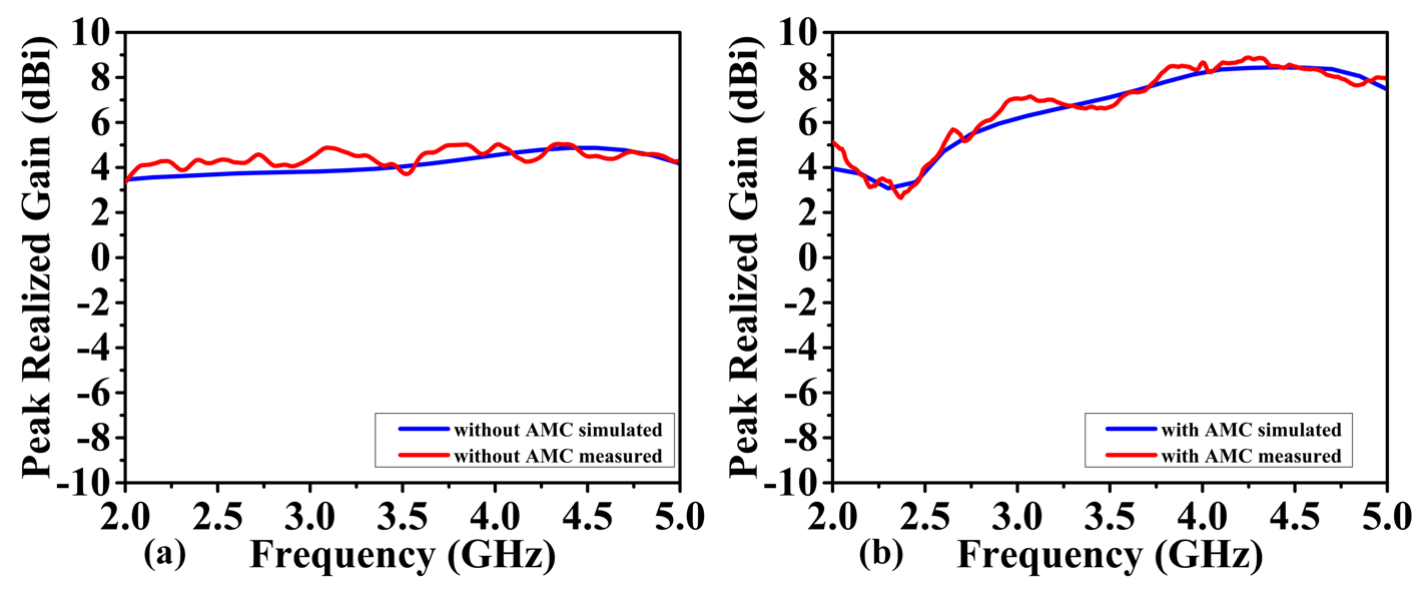
Realized gain of the proposed antenna measured and simulated: (**a**) without AMC, (**b**) with AMC.

**Figure 14:**
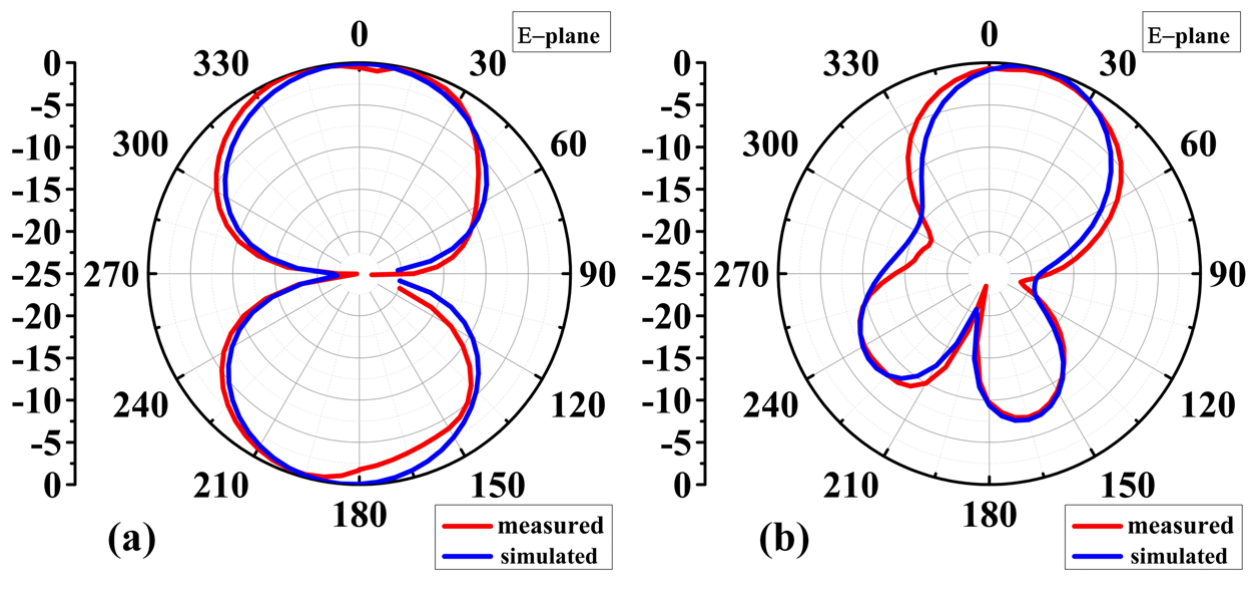
Simulation and measurement of 2D normalized E-plane radiation patterns of the proposed sensor at the resonant frequency: (**a**) without AMC, (**b**) with AMC.

**Figure 15:**
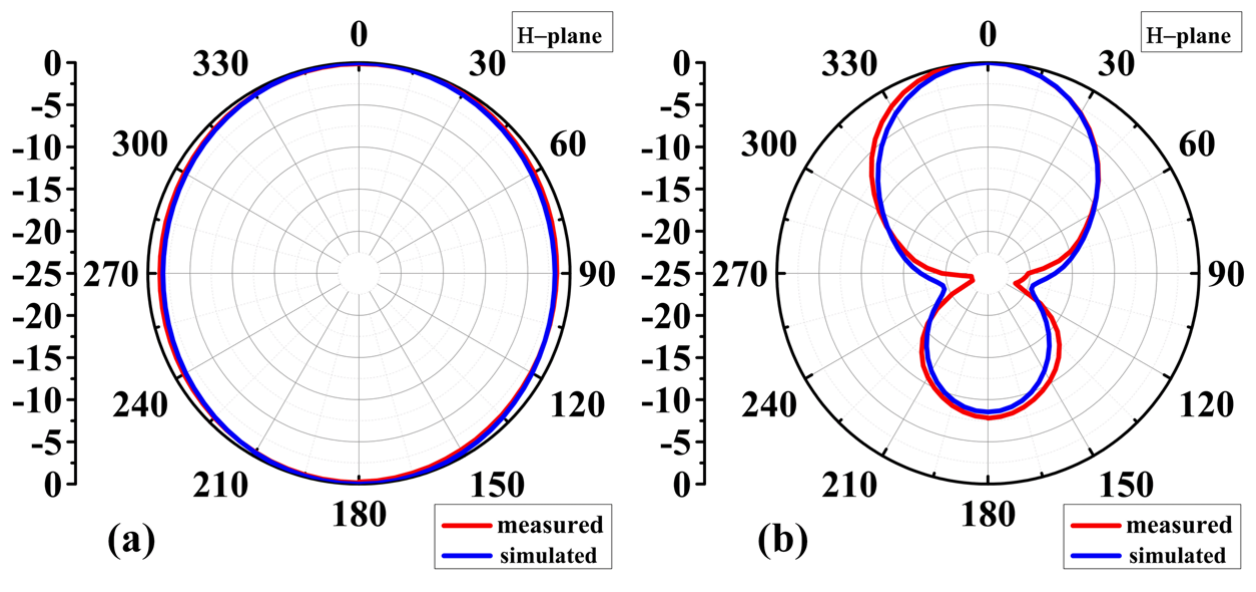
Simulation and measurement of 2D normalized H-plane radiation patterns of the proposed sensor at the resonant frequency: (**a**) without AMC, (**b**) with AMC.

Figures 12 and 13 show the antenna reflection and gain characteristics with and without the AMC layer, respectively. The agreement between the simulation and measurement data is excellent. Without AMC, the measured first resonant frequency and the resonance depth are 2.9 GHz and –41 dB, respectively, whereas the simulated figures are 2.75 GHz and –29 dB. Furthermore, without AMC, the measured values ​​of the second resonant frequency and resonance depth are 4.751 GHz and –29 dB, respectively, whereas the simulated values ​​are 4.75 GHz and –21 dB, as shown in Fig. 12a. For the complete sensor (with the AMC layer attached to the antenna), as shown in Fig. 12b, the measured values ​​of 4.51 GHz and –24 dB versus the simulated resonant frequency of 4.56 GHz and reflection coefficient of –50 dB. The observed discrepancies between EM simulations and experimental results can be attributed to the effect of the SMA connector, assembly errors, and manufacturing imperfections. Due to their smooth, flat surfaces, metallic structures are susceptible to surface waves, allowing electromagnetic waves to propagate between the metal and the surrounding environment^53^. Surface waves in microwave streams radiate vertically when they come into contact with curved, deformed, or uneven surface patterns^54^. The proposed AMC corrects the waves that may be wasted at extreme angles and controls the sensor's emission pattern in a reasonable direction. As a result, the addition of AMC increases the antenna gain^55^. Referring to Fig. 12a, the gain at the second resonant frequency in the absence of the AMC is 4.8 dBi (simulation) and 4.7 dBi (measurement). Figure 13b displays the antenna's realized gain while using the AMC. At the resonant frequency, the gain increases to 8.4 dBi (simulation) and 8.47 dBi (measurement) when AMC is included. Thus, for simulation and measurement, the gain augmentation due to AMC can reach up to 3.6 dBi and 3.77, respectively. Figures 14 and 15 show the radiation patterns in the E and H planes of the proposed sensor, respectively. The H-plane is defined as the plane perpendicular to the base feed line of the antenna, while the E-plane is perpendicular to the antenna substrate and aligned with the feed line. Figures 14a and 15a show the arrangement without AMC, while Figs. 14b and 15b show the entire sensor (antenna + AMC). As it can be observed, the employment of the AMC leads to creating a directional radiation pattern. The latter is essential to the effectiveness of MWI, especially when it comes to facilitating identification of breast, brain, and lung cancer.

## Breast cancer diagnosis

**Figure 16:**
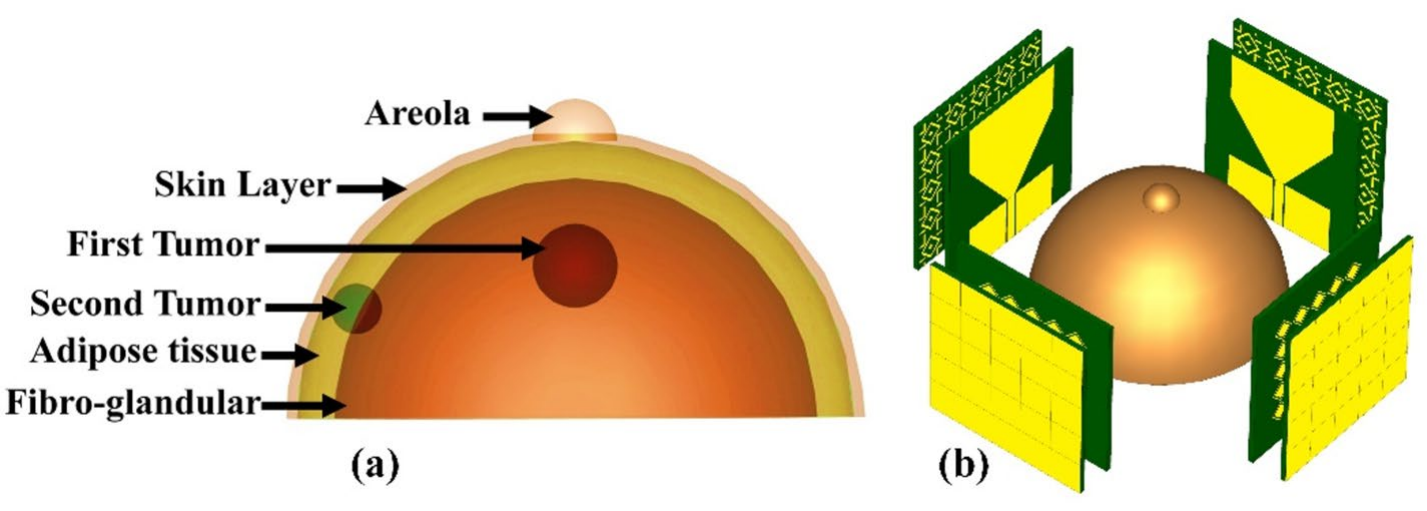
Breast cancer detection using the suggested sensor: (**a**) malignant breast phantom; (**b**) MWI system simulation configuration.

**Figure 17:**
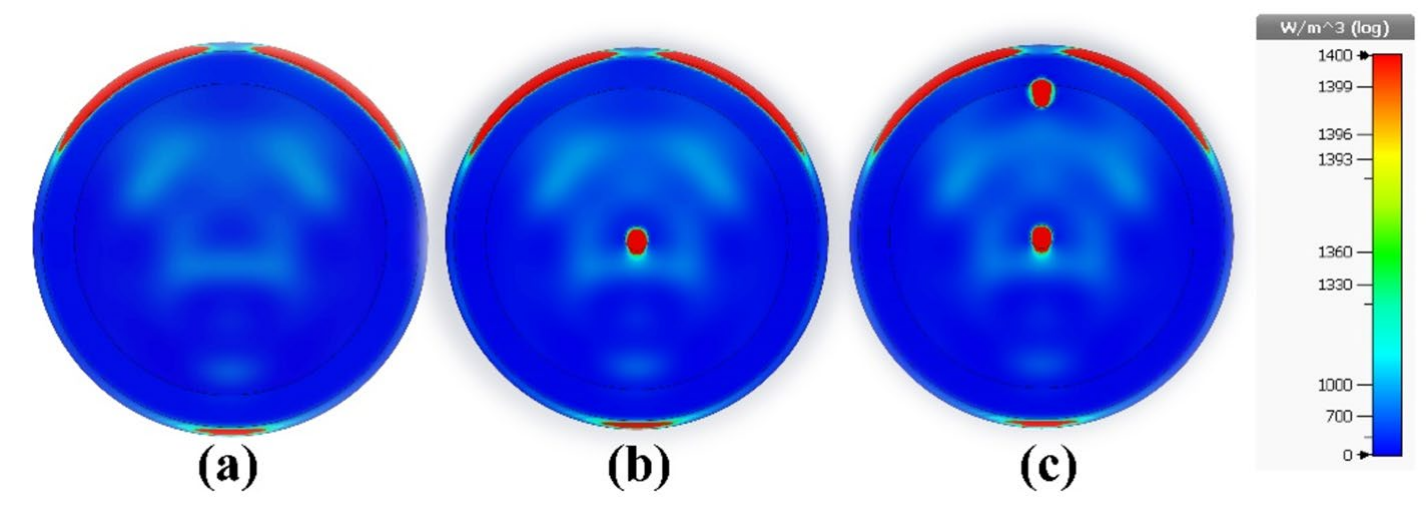
Microwave imaging results at 4.562 GHz: (**a**) normal breast; (**b**) non-invasive (early-stage) breast cancer with one tumor; and (**c**) invasive breast cancer with two tumors.

**Figure 18:**
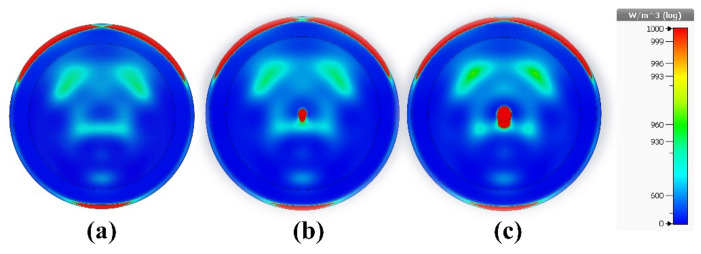
Microwave imaging results at 4.562 GHz to examine tumor volume change in: (**a**) a healthy breast; (**b**) the fourth case; and (**c**) the fifth case.

Radar-based microwave imaging for early detection of breast cancer uses antennas to transmit and receive microwave signals within the breast. A detection is based on different electrical properties of cancerous and healthy tissue. This process involves the targeted transmission of microwave radiation, resulting in scattering or absorption of the signal. The reconstructed images are color-coded for display, with red indicating a high dielectric constant and conductivity associated with tumor tissue and blue or green indicating healthy adipose or muscle tissue. This approach exploits the contrast in dielectric properties between healthy and tumor tissue, allowing microwave signals at specific frequencies to penetrate biological tissues. Tumor tissues, especially at early stages, exhibit pronounced dielectric contrast due to higher water content, altered cellular structure, and changes in blood perfusion. Color-coded images depict tissue dielectric properties, helping healthcare professionals identify potential abnormalities. The performance of the antenna proposed in this study in the frequency and time domains makes it suitable for microwave breast imaging and tumor identification. We use a five-layer breast model for demonstration purposes, as shown in Fig. 16a. Four copies of the sensor for breast tumor detection are shown in Fig. 16b. Many tumors demonstrate the unintentional growth and spread of cancer cells. Table 2 shows the individual characteristics of these levels. With survival rates of 99%^7^ and 97%^56^, the study sought to exploit signals scattered in the early stages of breast cancer to distinguish between newly malignant breasts and healthy breasts. The sensor was tested on three different cases: pure breast phantom, non-invasive (early stage) cancer with 2 mm-radius tumors, and invasive cancer with multiple tumors. The first tumor was found in fibro-glandular tissue, whereas the second tumor was found in fibro-glandular and fatty tissue. The distance between the two tumors was 23 mm, with the radius of the second tumor being 2.5 mm larger. Tumors increase the concentration of electric and magnetic fields, making it easier to detect and locate tumors in breast tissue, as shown in Fig. 17. In the fourth and fifth cases, a normal breast is shown in Fig. 18a; also, the size of the tumor was studied, from a small tumor with a radius of 1.5 mm to a larger tumor with high risk, as shown in Fig. 18b. Subsequently, a tumor is studied with a radius of 2.5 mm, as explained in Fig. 18c. By analyzing backscatter signals, our proposed system accurately detects tumors, making it a good candidate for microwave breast imaging.

**Table 2:** Dielectric properties of the human breast tissues with tumor and malignant cells.

Tissue type	Relative permittivity^5,57,58^	Electric or effective conductivity (σ_eff_) (S/m) ^5,57,58^	Density (kg/m^3^)^5,58^	Thermal conductivity (W/K m)^30,59^	Specific heat capacity (kJ/K kg)^30,59^	Diffusivity (m^2^/s)
Areola	36.7	2.34	1109	0.52	3.92	1.19615 × 10^−7^
Skin	36.7	2.34	1109	0.52	3.92	1.19615 × 10^−7^
Adipose tissue	4.84	0.262	911	0.23	1.9	1.32879 × 10^−7^
Fibro-glandular	20.1	0.5	1035	0.51	3.9	1.26347 × 10^−7^
Tumor	67	4	1085	0.55	3.75	1.35177 × 10^−7^

## Lung cancer diagnosis

**Figure 19:**
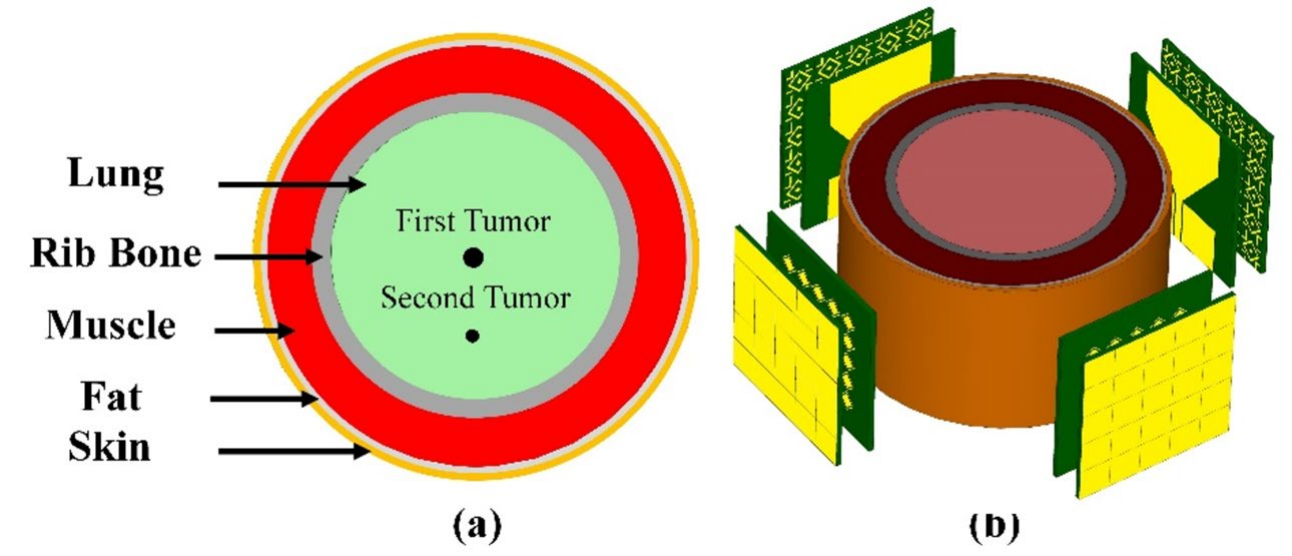
Lung cancer detection using the suggested sensor: (**a**) cancerous lung phantom; (**b**) MWI system simulation setup.

**Figure 20:**
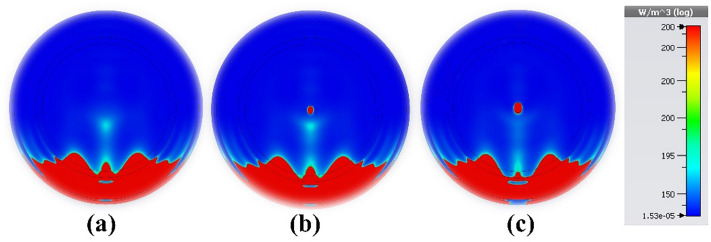
Microwave imaging technique was utilized at 4.562 GHz to examine tumor volume change in: (**a**) first case (healthy lung); (**b**) the second case; and (**c**) the third case.

**Figure 21:**
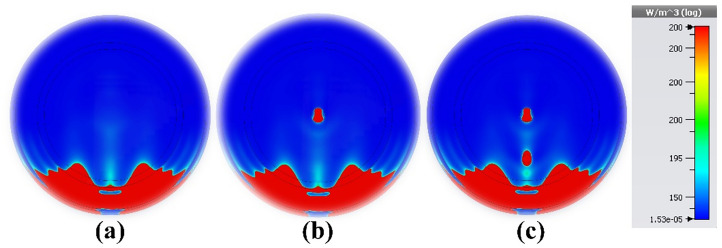
Microwave imaging results at 4.562 GHz: (**a**) healthy lung; (**b**) fourth case; and (**c**) fifth case (brain cancer with two tumors).

The properties of the proposed sensor also make it suitable for lung cancer detection in the MWI regime, in addition to its ability to detect undesirable tissue alterations using breast imaging. The five layers that make up the lung model used in this study are shown in Fig. 19a. The material properties of the stages are summarized in Table 3. As shown in Fig. 19b, four copies of the proposed sensor are illustrated as a part of the MWI system for lung tumor detection. The growth and spread of malignant cells are indicated by the abundance of tumors. Using microwave imaging, the electrical properties of newly infected tumors or malignant cells can be compared with the electrical properties of normal, healthy cells and tissues. Tumors increase the electric field strength, making them easier to detect and localize in lung tissue. Also, tumors are associated with unique microwave interactions, affecting signal absorption, reflection, or scattering. Despite the challenges in signal transmission, image reconstruction and resolution, the opportunity lies in utilizing the unique dielectric properties of the tumor, the electrical and field properties of the sensor (high gain, ultra-wide bandwidth and directional radiation pattern), as well as in confocal microwave imaging techniques. Tumor sizes in four early-stage lung cancer cases were carefully examined. The results show that a wide range of malignancy sizes can be detected using MWI, with the supplied sensor forming a key part of the imaging system. These range in size from small, localized tumors to larger, more aggressive tumors. In the first case, it was a pure lung phantom, as shown in Fig. 20a. In the second case, a tumor with a radius of 2 mm was detected, as shown in Fig. 20b. Figure 20c shows the tumor in the third case, with a radius of 3 mm. The fourth case was an early-stage cancer with a tumor radius of 4 mm, and the fifth case was a malignancy with multiple tumors. Also, a pure lung phantom is shown in Fig. 21a; the second tumor is 20 mm away from the first tumor and has a radius of 3 mm, as shown in Fig. 21b and c, respectively.

**Table 3:** Electrical properties of the distinct tissues in the cylindrical lung model^60,61^.

Tissue	Electric or effective conductivity (σ _eff_) (S/m)	Relative permittivity (ε_r_)
Tumor	5.454	61.8
Lung	1.21	19.7
Rib Bone tissue	1.28	17.2
Muscle tissue	2.74	51.2
Fat	0.453	10.4
Skin	2.15	36.8

## Brain cancer diagnosis

**Figure 22:**
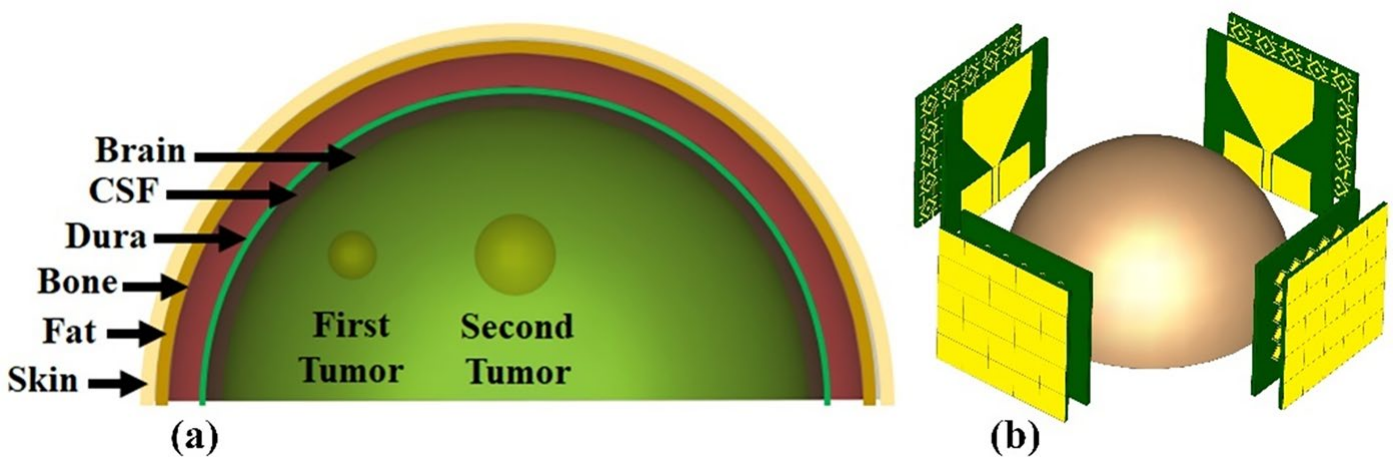
Application of the proposed sensor for brain cancer detection: (**a**) cancerous brain phantom; (**b**) MWI system simulation setup.

**Figure 23:**
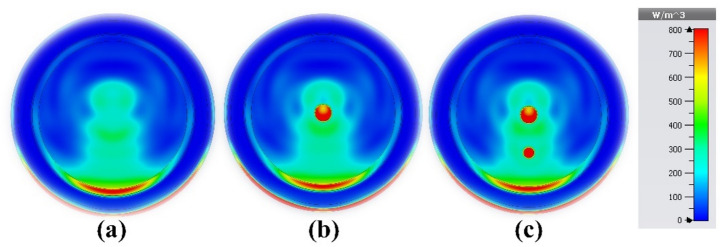
Microwave imaging findings at 4.562 GHz: (**a**) healthy brain; (**b**) early-stage brain cancer with one tumor; and (**c**) brain cancer with two tumors.

**Figure 24:**
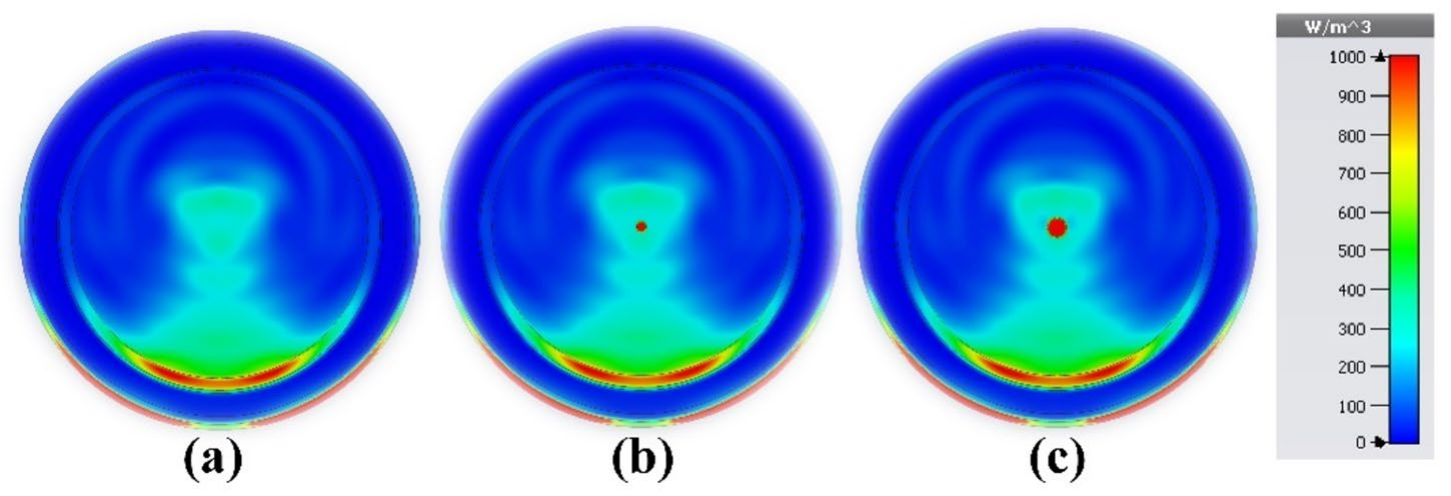
Microwave imaging results at 4.562 GHz to examine tumor volume change in: (**a**) a healthy brain; (**b**) the first case; and (**c**) the second case.

**Figure 25:**
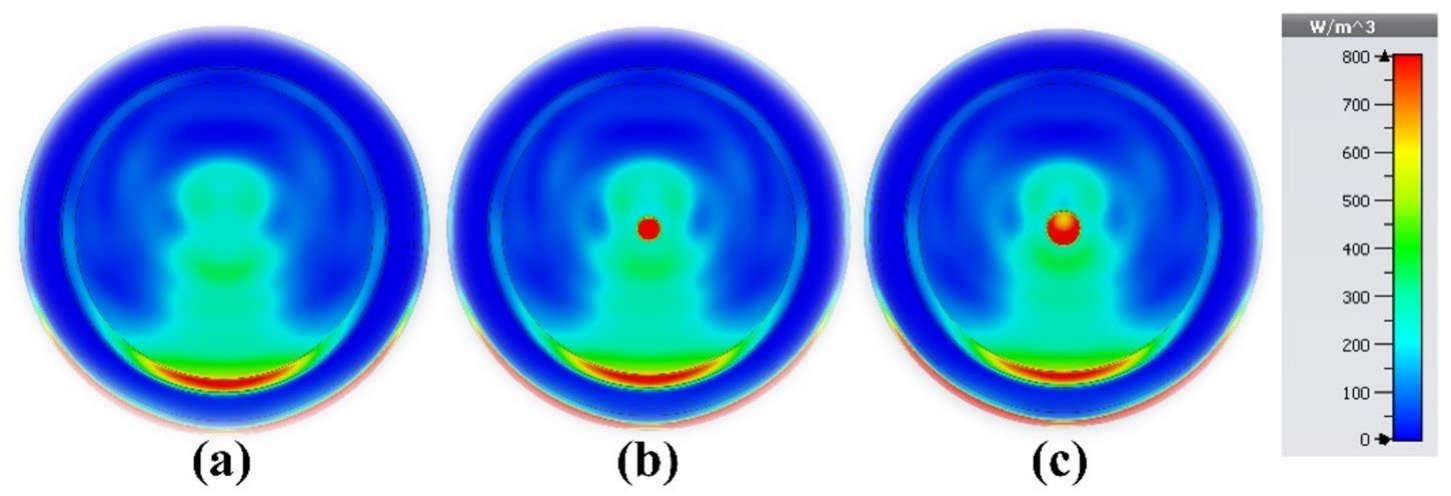
Microwave imaging results at 4.562 GHz used to investigate tumor volume change in: (**a**) a healthy brain; (**b**) the third case; and (**c**) the fourth case.

As shown in Fig. 22a, the brain model used in this study consists of seven different layers. The material characteristics of the strata are listed in Table 4. The MWI system for brain tumor detection includes four antennas, an electromagnetic wave generator, a collection system, and a processor, as shown in Fig. 22b. The radiation delivered to the test head is altered due to the presence of the target. After receiving a signal containing target information, the processor will extract the data as an image. Post-processing techniques are applied to improve the results. Interested readers may find comprehensive explanations of image rendition techniques the literature^62,63^. For the purpose of verification, the proposed sensor was used in three scenarios: a pure brain phantom, an early-stage cancer with a tumor radius of 4 mm, and an aggressive disease with multiple tumors. Featuring a radius of 3 mm, the second tumor is located 15 mm away from the first tumor, as shown in Figs. 23a–c, respectively. Tumors increase the strength of the E-field and H-field, as shown in Fig. 23, making it easier to identify and locate them in the brain tissue. The presence of multiple tumors indicates the growth and metastasis of cancer cells. The electrical properties of newly infected tumors or malignant cells can be compared with normal, healthy cells and tissues using microwave imaging. The tumor size of four early-stage brain cancer cases was studied in detail. The results demonstrate a wide range of malignancy sizes, from small, localized tumors to larger, more aggressive tumors. These results emphasize the need for early detection and appropriate treatment for brain cancer patients. A normal brain is shown in Fig. 24a; as shown in Fig. 24b, a tumor with a radius of 1 mm was found in the first case. Figure 24c shows the tumor in the second case, with a radius of 2 mm. Also, a normal brain is shown in Fig. 25a; the third case shows a tumor with a radius of 3 mm, as shown in Fig. 25b. Finally, as indicated in Fig. 25c, a tumor of a radius of 4 mm was found in the fourth case. These results corroborate the ability of the proposed sensor to detect and discriminate tumors of various sizes. This property facilitates classification of the different stages and symptoms of cancer for diagnostic purposes, simplifying the process of choosing the most effective treatment. Overall, the results show that the provided sensor holds great promise in accurately identifying medical conditions.

**Table 4:** Hemispheric head model's distinct tissues' electrical properties^45,52,60^.

Tissue	Electric or effective conductivity (σ_eff_) (S/m)	Relative permittivity (ε_r_)
Brain	1.29	43.22
Cerebrospinal fluid (CSF)	2.3	70.1
Dura	0.9	46
Bone (skull)	0.03	5.6
Fat	0.04	5.54
Skin	0.73	45
Tumor	7	55

## Benchmarking

The proposed sensor stands out from existing MWI technologies through key advantages that enhance its performance and applicability in medical diagnostics. The integration of a metamaterial layer-based lens and an artificial magnetic conductor (AMC) separate layer contributes significantly to improved directional radiation pattern, high realized gain, and enhanced sensitivity in detecting abnormalities like tumors. These features enable precise tumor size discrimination and facilitate early-stage diagnosis, aligning with the critical requirements for effective medical testing using MWI. Moreover, the sensor's competitive performance, as validated through extensive experimental testing and simulation-based studies, demonstrates its superiority in critical characteristics such as gain, pattern directivity, and impedance matching when compared to state-of-the-art designs reported in recent literature. This comprehensive evaluation showcases the distinct advantages of our sensor, positioning it as a robust solution for high-quality MWI applications and contributing significantly to advancements in medical imaging technologies. Here, a detailed comparison between the proposed sensors and state-of-the-art sensor designs published in the recent literature is carried out. Tables 5 and 6 show data for sensors designed to detect breast cancer and brain cancer, respectively. Unfortunately, there is no accurate data concerning the electrical and field performance for sensors used to identify lung cancer. That said, the scope of the comparison presented in Tables 5 and 6 is sufficiently comprehensive to ensure meaningful assessment. The considered characteristics include antenna size, operating frequency range, substrate material parameters, and antenna field characteristics (especially, realized gain). As it can be seen, the proposed sensor has significantly higher gain than the majority of the benchmark antennas. Another feature that sets it apart from the models in Tables 5 and 6 is its highly directional radiation pattern. Additionally, the extensive investigations presented in "Breast cancer diagnosis", "Lung cancer diagnosis", and "Brain cancer diagnosis" sections supports the adaptability of the device. It is demonstrated that our sensor can be successfully used in the diagnosis of many conditions (here, breast cancer, lung cancer, and brain tumors), while the majority of reference models are created for a single purpose (e.g., detecting breast cancer). However, the practical implementation of our MWI-enabled cancer detection sensor faces several limitations. Integration challenges arise from the sensor's size, and the costs of fabrication and deployment must be considered for scalability. Additionally, potential safety concerns with microwave radiation necessitate strict adherence to regulatory guidelines. Collaborative efforts with medical professionals and industry partners are essential to optimize size, cost, and safety standards, enhancing the sensor's practicality and effectiveness in real-world medical settings, thereby improving early cancer detection and patient outcomes.

**Table 5:** Comparison of suggested sensor for breast cancer detection with state-of-the-art antennas reported in the literature.

References	Structure size (mm^2^)	Substrate	Frequency range (GHz)	Gain (dBi)	Year published
^41^	40 × 40	FR4	3–11	7.06	2018
^34^	51 × 42	Rogers RT/duroid 5870	2–7.5	9.5	2019
^36^	21.44 × 23.53	FR4	3–12	5.76	2019
^38^	40 × 40	FR4	2–11	7.2	2019
^39^	40 × 40	FR4	2–11	7.2	2019
^37^	42 × 41	Rogers RT 5880	2–11	5.40	2020
^5^	20 × 19	FR4	2–12	5	2022
^64^	80 × 61	felt	4–15	7.56	2022
This work	50 × 50	FR4	2–5	8.47	–

**Table 6:** Comparison of the proposed sensor and state-of-the-art antennas for brain cancer detection.

References	Structure size (mm^2^)	Substrate	Frequency range (GHz)	Gain (dBi)	Year published
^51^	70 × 15	Rogers 3003	0–3.5	3	2016
^42^	80 × 20	FR4	0.75–2.5	4.6	2016
^45^	31.68 × 31	Rogers R03003	3–10	6.77	2018
^43^	50 × 60	Rogers RO4350B	2–3	2.45	2019
^50^	59 × 59	Rogers R04003C	0.6–1.4	3.1	2019
^52^	50 × 44	Rogers RO4350B	1.5–4	5.65	2020
^49^	50 × 40	Rogers RT5880 and RO4350B	1.3–3.3	6.67	2022
This work	50 × 50	FR4	2–5	8.47	–

## Future perspective

The ability to integrate our innovative metamaterial-based sensor with existing microwave imaging (MWI) systems provides an exciting direction for future research. This integration can provide several benefits, including enhanced imaging capabilities, improved diagnostic accuracy, and expanded applicability in clinical settings. By integrating our sensors into existing MWI systems, we can leverage the unique characteristics of both technologies to create a more comprehensive and efficient imaging platform. This collaboration has the potential to advance early cancer detection and improve patient outcomes by providing clinicians with advanced tools for accurate diagnosis and treatment planning. Future research in this area will focus on seamlessly integrating our sensor with MWI systems, optimizing performance, and validating its clinical utility through validation protocols, and strict inspection.

## Conclusions

This paper introduced a novel sensor to operate in microwave imaging (MWI) systems, where high realized gain, directional radiation pattern, and wide impedance bandwidth are the key performance requirements. The aforementioned qualities have been realized through meticulous design of the sensor architecture. The two sensing components are deployed on different substrate layers. The basic antenna is developed using an array of metamaterial (MTM) unit cells and a coplanar microstrip patch fed by a waveguide. Subsequently, an artificial magnetic conductor (AMC) structure is installed and allocated in parallel to the main radiator. Furthermore, the analysis of permeability and permittivity show that the proposed AMC exhibits negative epsilon (ENG) characteristics at the resonance point of the antenna. In addition, transmittance, absorption, and reflectivity are also tested. The mentioned properties contribute to realization of directional radiation pattern and high realized gain of the sensor, which are crucial for improving the sensitivity and quality of medical tests using MWI. This, in turn, facilitates identification of abnormalities at the early stages of diagnosis and the selection of the best treatment method. Experimental validation has been performed using the sensor prototype fabricated on FR4 substrate showing excellent agreement between EM simulations and measurements. At the resonant frequency of 4.56 GHz, the measured gain is 8.5 dBi. The presented antenna performs comparably to the state-of-the-art designs documented in the literature. Sensor integration and testing of the MWI system demonstrated its suitability for real-world imaging applications requiring focused radiation, high gain, and wide bandwidth. Our sensor can be used to diagnose a variety of diseases, such as brain tumors, lung cancer, and breast cancer. This has been demonstrated by extensive simulation testing performed using the computational model of the MWI system, which integrates four instances of the proposed sensor along with breast, lung, and brain phantoms. One of its main features is that the proposed sensor allows precise discrimination of tumors of different sizes due to the enhancement gain and directional radiation pattern. For this reason, it is a strong candidate for high-end, versatile microwave imaging systems. Finally, an in-depth comparative study with state-of-the-art designs reported in the recent literature was performed, confirming that the proposed sensor exhibits superior properties, especially in terms of characteristics. such as gain, pattern directivity, and impedance matching, which are essential for high-quality MWI.

## Data Availability

The datasets used and/or analyzed during the current study available from the corresponding author on reasonable request.
